# Characteristics of the Passive Muscle Stiffness of the Vastus Lateralis: A Feasibility Study to Assess Muscle Fibrosis

**DOI:** 10.3390/ijerph18178947

**Published:** 2021-08-25

**Authors:** Akifumi Maeda, Maito Yamagishi, Yuta Otsuka, Takayuki Izumo, Tomohiro Rogi, Hiroshi Shibata, Masahiro Fukuda, Takuma Arimitsu, Yosuke Yamada, Naokazu Miyamoto, Takeshi Hashimoto

**Affiliations:** 1 Faculty of Sport and Health Science, Ritsumeikan University, Shiga 525-8577, Japan; gr0318vf@ed.ritsumei.ac.jp (A.M.); 0110.maito.0110@gmail.com (M.Y.); arimitsu@hachinohe-u.ac.jp (T.A.); 2 Suntory Global Innovation Center Ltd., Research Institute, Kyoto 619-0284, Japan; 3 Institute for Health Care Science, Suntory Wellness Ltd., Kyoto 619-0284, Japan; Yuta_Otsuka@suntory.co.jp (Y.O.); Takayuki_Izumo@suntory.co.jp (T.I.); Tomohiro_Rogi@suntory.co.jp (T.R.); Hiroshi_Shibata@suntory.co.jp (H.S.); 4 Fukuda Clinic, Osaka 532-0003, Japan; fukuda@drmog.jp; 5 Undergraduate Department of Human Health, Faculty of Health Care, Hachinohe Gakuin University, Aomori 031-8588, Japan; 6 National Institutes of Biomedical Innovation, Health and Nutrition, Tokyo 162-8636, Japan; yamaday@nibiohn.go.jp; 7 Graduate School of Health and Sports Science, Juntendo University, Chiba 270-1695, Japan; n-miyamoto@juntendo.ac.jp

**Keywords:** extracellular matrix, sarcopenia, thigh muscle, ultrasound shear wave elastography

## Abstract

Skeletal muscle fibrosis occurs with aging and has been suggested to impair muscle performance, thereby decreasing quality of life. Recently, muscle stiffness, a surrogate measure of muscle fibrosis, was noninvasively quantified as the shear modulus using ultrasound shear wave elastography (SWE) in humans. We aimed to investigate thigh muscle stiffness in females and males, respectively, across a broad range of ages by using SWE. Eighty-six community-dwelling Japanese people who were aged 30 to 79 years and did not regularly exercise participated in this study. The vastus lateralis (VL) shear modulus was measured at three different knee joint angles: full extension, 90° of flexion, and full flexion. There were no significant main effects of sex or age on the VL shear modulus in full extension or 90° of flexion of the knee. However, the VL shear modulus in knee full flexion was significantly smaller in females than in males and increased with age from 47.9 years. The results suggest that the accelerated increase in VL stiffness that occurs after an individual passes their late 40s may be an important therapeutic target for developing effective treatments and programs that preserve and improve quality of life.

## 1. Introduction

Skeletal muscle fibrosis, an excessive accumulation of extracellular matrix (ECM) components, occurs with aging [1,2]. Advanced muscle fibrosis with aging leads to impaired muscle regeneration [3]. Furthermore, muscle fibrosis and the associated increase in muscle stiffness are suggested to impair mobility and exercise capacity, thereby decreasing the quality of life of elderly people [2]. Thus, muscular conditions in relation to fibrosis need to be assessed quantitatively to develop effective treatments and programs that preserve and improve quality of life.

There is growing evidence indicating that ECM components are primarily responsible for muscle stiffness [4,5]. For example, animal experiments have revealed that muscle bundles, which consist of several muscle fibers and contain ECM components, displayed more than 5-fold stiffness than individual muscle fibers and muscle fiber groups, excluding ECM components [6]. Similarly, human experiments have shown that the stiffness of muscle bundles is more than 15 times higher than that of single muscle fibers, although ECM components account for only approximately 5% of the muscle bundle cross-sectional area [7]. Furthermore, the contributions of ECM components to muscle stiffness depend on to what extent the muscle is stretched; the contributions of ECM components are high when the muscle is tensioned and stretched [8]. Collectively, it is reasonable to presume that muscle stiffness is a surrogate measure of an excessive accumulation of ECM components, i.e., muscle fibrosis, especially when muscle stiffness is evaluated in stretched positions. Indeed, a recent animal study demonstrated that muscle stiffness was well correlated with muscle fibrosis quantified histologically and suggests that passive stretch-induced stiffening notably reflected skeletal muscle fibrosis [9].

Until several years ago, age-related changes in muscle stiffness have rarely been studied in humans because it was difficult to noninvasively and directly assess muscle stiffness in humans in vivo. However, currently, the stiffness of in vivo human muscles can be noninvasively quantified as the shear modulus (expressed in the unit of Pascal) using ultrasound shear wave elastography (SWE). Several human studies have used this technique to investigate the effect of age on muscle stiffness and reported decreases [10,11], no changes [10,12], and increases [13] in stiffness in elderly adults compared with young adults. Note that in these studies reporting decreases or no changes in muscle stiffness in elderly adults, muscle stiffness was assessed in positions in which the muscles were relatively short (i.e., less stretched), such as a lying (fully knee extended) position for the quadriceps femoris and a plantarflexed position for the triceps surae. An animal study showed that muscle stiffness increases with age when muscle stiffness is measured in stretched rather than shortened positions [14]. In this context, only one recent study [13] showed that age-related changes in passive muscle stiffness in the human medial gastrocnemius (GM) can be observed in stretched (i.e., dorsiflexed) positions but not in neutral or shortened (i.e., plantar flexed) positions. However, it remains unclear whether this finding holds true for other muscles, especially those that exhibit significant functional decline with aging, and at what age the changes in muscle stiffness occur.

In addition to age, sex also influences muscle stiffness; females exhibit lower muscle stiffness values than males [15]. The sex difference in muscle stiffness is likely due, at least in part, to the influence of sex hormones such as estrogen because estrogen alters the structural and mechanical properties of collagenous tissues [16]. Based on this finding, we hypothesized that the sex difference in muscle stiffness disappears after the fifth or sixth decade, when most females experience menopause. To the best of our knowledge, only a few studies have examined the effects of both age and sex on muscle stiffness with the biceps brachii in a relatively stretched position [17,18]. Muscle function generally declines with aging, especially in thigh muscles [19]. Nevertheless, no studies have yet addressed the interactive effects of age and sex on the muscle stiffness of thigh muscles.

The objectives of the present study were (1) to examine whether thigh muscle stiffness increases with age; (2) to identify at what age the increase in muscle stiffness occurs (if any); (3) to prove our hypothesis that the age-related increase in thigh muscle stiffness, an index of excessive accumulation of ECM components, can be observed only in a stretched position, but not in a shortened position, of the muscle; and (4) to test our second hypothesis that greater muscle stiffness in males than in females disappears after the fifth or sixth decade of life. To achieve these objectives, we investigated thigh muscle stiffness in positions in which the muscle is both shortened and lengthened in females and males, respectively, across a broad range of ages using ultrasound SWE.

## 2. Materials and Methods

### 2.1. Subjects

For this study, we recruited community-dwelling Japanese people from Osaka who were aged 30 to 79 years and were not regularly exercising. The exclusion criteria were the presence of disease in locomotor organs; the presence of cardiovascular disease affecting the exercise intervention; a history of severe disorders or clinically significant systemic diseases; an irregular lifestyle such as a night shift worker; a high level of alcohol consumption, which is more than 60 g of alcohol per day; smoking; the consumption of drugs or supplements that affect efficacy evaluations; and the presence of any medical condition judged by the medical investigator to warrant exclusion. Nursing mothers, pregnant women, and women of child-bearing potential were excluded. All subjects provided written informed consent before participation. The Ethics Committee of the Fukuda Clinic Research (approval no. IRB-20180317-7) and Ritsumeikan University (approval no. BKC-2017-084) approved the study protocol, which was performed in compliance with the Declaration of Helsinki.

### 2.2. SWE Measurements

**Figure 1:**
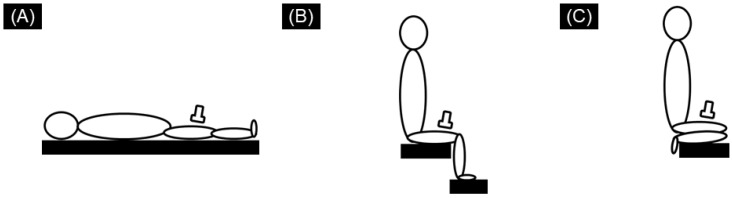
Schematic representation of the subject’s posture for ultrasound shear wave elastography (SWE) measurement. The vastus lateralis (VL) shear modulus was measured at the following three different knee joint angles: knee full extension in the supine position (**A**), 90° of knee flexion in the seated position (**B**), and knee full flexion in the seated position (with the right heel in contact with the hip) (**C**).

An ultrasound SWE apparatus (Aixplorer version 12, Supersonic Imagine, France), coupled with a linear array probe (SL10-2), was used to measure the shear modulus of the right VL. The VL shear modulus was measured at the following three different knee joint angles: (1) knee full extension in the supine position, (2) 90° of knee flexion in the seated position, and (3) knee full flexion in the seated position (with the right heel in contact with the hip) (Figure 1). The SWE measurements were performed in this order. In each position, the ultrasound probe was positioned at 50% of the thigh length (measured from the greater trochanter to the popliteal crease) according to a previous study [20]. The probe orientation was adjusted to identify fascicles within the ultrasound B-mode image in each position. Care was taken to not press or deform the muscles during the scan. The subjects were requested to completely relax the leg throughout the examination, although the muscle activity was not checked during SWE measurements. The subjects were not allowed to engage in warm-up or stretching exercises prior to the examination. The images were acquired after ensuring a stable color distribution of SWE mapping. The probe location was adjusted slightly prior to image acquisition if a defocused image with a large variation in SWE color mapping was observed. Measurements were performed three times in approximately 10 s in each position. Each measurement position was held for approximately 1–2 min before three SWE images were acquired. The room temperature for all measurements was kept constant at 25 ± 1 °C to minimize any potential temperature-induced changes in tissue stiffness.

The SWE data were analyzed using the software included with the ultrasound apparatus. The mean shear modulus was calculated over the region of interest (ROI) that was as large as possible with the exclusion of other tissues (e.g., aponeurosis and subcutaneous fat) and artifacts (e.g., unnaturally void areas), depending on the subject and position. We confirmed that no pixel reached the saturation limit of the SWE apparatus. The average value of the three measurements taken in each position was used for further analyses. The coefficients of variation of three measurements were 3.2 ± 2.4%, 1.4 ± 0.9%, and 1.0 ± 0.7%, with intraclass correlation coefficients of 0.978, 0.997, and 0.998, for knee full extension, 90° of knee flexion, and knee full flexion positions, respectively.

### 2.3. Statistical Analysis

The normality of the VL shear modulus in each position was confirmed using the Shapiro–Wilk normality test. Then, for the data in each position, two-way analysis of variance (ANOVA) (age [30s, 40s, 50s, 60s, 70s] × sex [female, male]) was performed using SPSS (version 26, IBM, Armonk, NY, USA). When a main effect of sex was found, an unpaired *t*-test was used to compare the females and males. When a main effect of age was found, we performed segmented regression analysis instead of multiple comparisons to identify at what age the change in muscle stiffness occurs, that is, to detect a breakpoint age of muscle stiffness using SegReg software (www.waterlog.info, accessed on: 14 March 2021). The significance level was set at α = 0.05.

## 3. Results

Forty-three female and 43 male adults aged 30 to 77 years participated in this study. At least seven females and seven males were included in each of the following age groups: 30–39, 40–49, 50–59, 60–69, and 70–79 years (Table 1). Most females aged 50–59, 60–69, and 70–79 years had experienced menopause.

**Figure 2:**
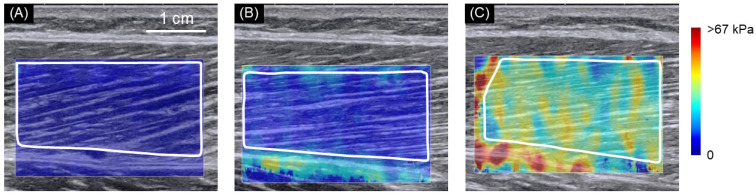
Typical examples of ultrasound shear wave elastographic (SWE) images. Typical examples of ultrasound SWE images with the knee fully extended in the supine position (**A**), the knee flexed to 90° in the seated position (**B**), and the knee fully flexed in the seated position (**C**). The colored region represents the shear wave speed map, with the scale to the right of the figure. The area drawn in solid white lines is the region of interest for the determination of shear modulus.

Figure 2 shows typical examples of ultrasound SWE images with the knee fully extended in the supine position (A), the knee flexed to 90° in the seated position (B), and the knee fully flexed in the seated position (C).

Table 2 shows the muscle shear modulus results for the VL stratified by sex and age group. For the VL shear modulus in knee full flexion, there were significant main effects of both sex and age, with no significant interaction effect of sex × age. For the VL shear modulus in knee full extension and 90° of flexion, two-way ANOVA showed there were no significant main effects or interaction effects of sex and age.

The post hoc tests revealed that the VL shear modulus was significantly smaller in females than in males. The segmented regression analysis revealed that the breakpoint age of the VL shear modulus in knee full extension was 47.9 (95% confidence interval: 44.5–50.8). Beyond the breakpoint, the VL shear modulus was estimated to increase by 0.5 kPa per year.

## 4. Discussion

In this study, an age-related increase in the VL shear modulus was observed in a stretched position (i.e., knee full flexion) but not in relatively shortened positions (i.e., knee full extension and 90° of flexion). This result is in line with previous results showing that an age-related increase in the passive muscle stiffness of the human GM can be observed in dorsiflexed positions but not in neutral or plantar flexed positions [13]. The joint angle specificity of age-related differences in muscle stiffness can be explained by the contribution of ECM. Muscle stiffness is strongly influenced by ECM components, such as the perimysium and endomysium [5]. However, the contributions of ECM components to muscle stiffness depend on to what extent the muscle is stretched; the contributions of ECM components are high when the muscle is tensioned and stretched [8]. Therefore, it is likely that muscle fibrosis, an excessive accumulation of ECM components, can be detected using ultrasound SWE only when muscle stiffness is measured in positions in which the muscle is stretched.

VL muscle stiffness increases with aging were observed in a stretched position probably due to an excessive accumulation of ECM components in the skeletal muscle, but the following two mechanisms may have been involved: (i) the absolute amount of ECM components increased, independent of the reduction in contractile components, and/or (ii) the relative amount of ECM components increased due to an age-related decrease in contractile components (muscular fibers), even though the absolute amount of ECM components remained unchanged. Regarding the first mechanism, the excessive accumulation of ECM components with aging has been reported in mice [1,2]. Furthermore, an increase in intramuscular connective tissues [21] and elevated serum C1q secretion with aging, which is suggested to activate the Wnt signaling pathway in muscles and lead to the development of muscle fibrosis [22], have also been observed in humans. For the second mechanism, age-related loss in muscle mass has been widely reported [23], and an acceleration in muscle loss after age 45 has also been reported [24]. However, since we cannot conclude which mechanism is more plausible on the basis of the results of this study, additional studies are necessary.

Importantly, we demonstrated that VL muscle stiffness in knee full flexion increased from 47.9 years of age. Although the excessive accumulation of ECM components has previously been reported to occur with aging [1,2], the present data suggest that age-related increases in ECM components may not be merely linear; rather, the changes may accelerate after a certain age. In a previous study that used ultrasound SWE, when the biceps brachii was stretched (i.e., elbow fully extended), muscle stiffness was observed to increase linearly after 60 years of age [17]. Another study reported that GM muscle stiffness measured in a stretched (i.e., ankle dorsiflexed) position was the highest in the older group (62 ± 3 years of age), followed by the middle-aged group (35 ± 3 years of age) and then the child group (7 ± 1 years of age) [13]. Although the findings of the previous and present studies are consistent in that muscle stiffness measured in stretched positions increases with age, the age at which muscle stiffness begins to increase is inconsistent between the previous and present studies; VL muscle stiffness in the present study started to increase from the late 40s, which is approximately 15 years earlier than that reported for the biceps brachii in a previous study [17]. This difference might reflect a site-specific increase in muscle stiffness. It has been reported that the loss of muscle mass after the age of 45 is more pronounced in the lower body than in the upper body [24], and a decrease in muscle mass in the thigh has been observed earlier than that at the whole body level [25]. This finding might support our aforementioned suggestion that the relative increase in ECM components caused by age-related decreases in contractile components might be related to the increase in muscle stiffness in a stretched position with aging. This idea is merely speculation at this moment, but these findings suggest that the age at which fibrosis begins to occur varies depending on the site within the body. Given that muscle function generally declines with aging, especially in thigh muscles [19], our finding implies that the accelerated increase in VL stiffness that occurs after an individual passes their late 40s may be an important therapeutic target for developing effective treatments and programs that preserve and improve quality of life.

In the present study, males showed higher muscle stiffness than females in the stretched position (but not in the relatively shortened (i.e., less stretched) positions). This result is consistent with those of previous studies on the GM in a young population [15]. The sex difference in muscle stiffness is reportedly due, at least in part, to the influence of sex hormones such as estrogen because estrogen alters the structural and mechanical properties of collagenous tissues [16]. Based on this finding, we hypothesized that the sex difference in muscle stiffness could not be observed after the fifth or sixth decade of life, when most females experience menopause. However, contrary to our hypothesis, our results showed a significant main effect of sex and no significant interaction effect of sex × age, indicating that muscle stiffness is higher in males than in females at all ages. Although serum hormone levels were not measured in this study, our findings suggest that the effect of the level of sex hormones decreasing due to menopause on muscle stiffness is negligibly small and that the cumulative effect of sex hormones over several decades prior to menopause in females is much larger.

## 5. Conclusions

Age-induced changes in skeletal muscle profiles have been reported to affect muscle performance, such as muscle strength and muscle power, more than muscle mass in elderly individuals [23]. In this study, we demonstrated an age-related increase in muscle stiffness in the VL in a stretched position in both females and males from 47.9 years of age, which indicates an increase in ECM components. Taken together, these reports and the present findings suggest that not only the accelerated decline in muscle mass but also the accelerated increase in muscle ECM components, starting from the age mid 40s onwards, may be responsible for corresponding declines in muscle performance parameters, such as muscle strength and muscle power. It is necessary to address the increase in the ECM components particularly in people who have passed their mid 40s to develop countermeasures against decreases in the quality of life in elderly people.

## Figures and Tables

**Table 1:** Characteristics of the participants.

	Age Group (years)	30–39	40–49	50–59	60–69	70–79
Female	*N*	9	9	9	7	9
	Age (years)	34.78 ± 3.01	45.11 ± 2.23	54.67 ± 2.49	65.29 ± 3.10	71.78 ± 2.20
	Height (cm)	158.71 ± 4.69	155.88 ± 4.07	155.41 ± 4.58	154.43 ± 2.71	151.94 ± 2.75
	Weight (kg)	51.22 ± 7.24	51.73 ± 4.23	53.80 ± 6.16	46.20 ± 2.64	51.27 ± 5.73
	BMI	20.30 ± 2.47	21.31 ± 1.85	22.30 ± 2.60	19.36 ± 0.77	22.19 ± 2.23
	Number of menopause	0	0	8	7	9
Male	*N*	9	9	9	9	7
	Age (years)	34.44 ± 2.79	43.89 ± 2.88	53.44 ± 2.71	65.56 ± 1.64	73.14 ± 2.53
	Height (cm)	175.40 ± 4.70	171.57 ± 4.36	169.97 ± 4.85	169.49 ± 6.28	165.31 ± 3.72
	Weight (kg)	68.10 ± 5.84	70.32 ± 9.00	69.66 ± 7.05	69.67 ± 9.57	63.51 ± 10.50
	BMI	22.19 ± 2.33	23.83 ± 2.29	24.09 ± 2.02	24.37 ± 4.04	23.12 ± 2.96

The values are expressed as the mean ± standard deviation. BMI = body mass index.

**Table 2:** Muscle shear modulus of the vastus lateralis (VL) of the females and males in each age group.

Age Group (years)		30–39	40–49	50–59	60–69	70–79	Two-Way ANOVA *p*-Value (Partial η^2^)		
							(Age)	(Sex)	(Age X Sex)
Muscle shear modulus (kPa)									
The knee fully extended									
	Female	4.16 ± 0.97	4.31 ± 1.41	4.66 ± 2.27	4.90 ± 1.15	4.28 ± 0.67	0.413 (0.050)	0.408 (0.009)	0.645 (0.032)
	Male	3.92 ± 0.83	4.01 ± 0.46	3.93 ± 0.54	4.58 ± 1.25	4.80 ± 0.97			
The knee flexed to 90°									
	Female	8.63 ± 1.82	7.40 ± 1.23	10.69 ± 6.12	8.42 ± 2.11	10.71 ± 6.96	0.128 (0.089)	0.272 (0.016)	0.876 (0.016)
	Male	7.93 ± 0.97	7.16 ± 0.83	9.15 ± 2.17	8.75 ± 2.24	8.81 ± 1.83			
The knee fully flexed									
	Female	43.02 ± 9.44	41.03 ± 10.23	45.66 ± 16.74	46.26 ± 12.76	51.68 ± 14.77	0.006 (0.170)	0.001 (0.145)	0.497 (0.043)
	Male	56.72 ± 14.93	46.38 ± 10.44	53.60 ± 10.46	51.52 ± 13.67	70.79 ± 16.59			

The values are expressed as the mean ± standard deviation. There were no significant main effects or interaction effects of sex and age on the VL shear modulus in knee full extension or 90° of flexion. There were significant main effects of both age and sex, with no significant interaction effect of sex × age in knee full flexion (two-way ANOVA, *p* < 0.05).

## References

[1] Wood L.K., Kayupov E., Gumucio J.P., Mendias C.L., Claflin D.R., Brooks S.V. (2014). Intrinsic Stiffness of Extracellular Matrix Increases with Age in Skeletal Muscles of Mice. J. Appl. Physiol..

[2] Mahdy M.A.A. (2019). Skeletal Muscle Fibrosis: An Overview. Cell Tissue Res..

[3] Stearns-Reider K.M., D’Amore A., Beezhold K., Rothrauff B., Cavalli L., Wagner W.R., Vorp D.A., Tsamis A., Shinde S., Zhang C. (2017). Aging of the Skeletal Muscle Extracellular Matrix Drives a Stem Cell Fibrogenic Conversion. Aging Cell.

[4] Gillies A.R., Lieber R.L. (2011). Structure and Function of the Skeletal Muscle Extracellular Matrix. Muscle Nerve.

[5] Purslow P.P. (1989). Strain-Induced Reorientation of an Intramuscular Connective Tissue Network: Implications for Passive Muscle Elasticity. J. Biomech..

[6] Meyer G.A., Lieber R.L. (2011). Elucidation of Extracellular Matrix Mechanics from Muscle Fibers and Fiber Bundles. J. Biomech..

[7] Lieber R.L., Runesson E., Einarsson F., Fridén J. (2003). Inferior Mechanical Properties of Spastic Muscle Bundles Due to Hypertrophic but Compromised Extracellular Matrix Material. Muscle Nerve.

[8] Gajdosik R.L. (2001). Passive Extensibility of Skeletal Muscle: Review of the Literature with Clinical Implications. Clin. Biomech..

[9] Martins-Bach A.B., Bachasson D., Araujo E.C.A., Soustelle L., Loureiro de Sousa P., Fromes Y., Carlier P.G. (2021). Non-Invasive Assessment of Skeletal Muscle Fibrosis in Mice Using Nuclear Magnetic Resonance Imaging and Ultrasound Shear Wave Elastography. Sci. Rep..

[10] Akagi R., Yamashita Y., Ueyasu Y. (2015). Age-Related Differences in Muscle Shear Moduli in the Lower Extremity. Ultrasound. Med. Biol..

[11] Yoshida K., Itoigawa Y., Maruyama Y., Saita Y., Takazawa Y., Ikeda H., Kaneko K., Sakai T., Okuwaki T. (2017). Application of Shear Wave Elastography for the Gastrocnemius Medial Head to Tennis Leg. Clin. Anat..

[12] Wang C.-Z., Li T.-J., Zheng Y.-P. (2014). Shear Modulus Estimation on Vastus Intermedius of Elderly and Young Females over the Entire Range of Isometric Contraction. PLoS ONE.

[13] Liu X., Yu H.-K., Sheng S.-Y., Liang S.-M., Lu H., Chen R.-Y., Pan M., Wen Z.-B. (2021). Quantitative Evaluation of Passive Muscle Stiffness by Shear Wave Elastography in Healthy Individuals of Different Ages. Eur. Radiol..

[14] Rosant C., Nagel M.-D., Pérot C. (2007). Aging Affects Passive Stiffness and Spindle Function of the Rat Soleus Muscle. Exp. Gerontol..

[15] Miyamoto N., Hirata K., Miyamoto-Mikami E., Yasuda O., Kanehisa H. (2018). Associations of Passive Muscle Stiffness, Muscle Stretch Tolerance, and Muscle Slack Angle with Range of Motion: Individual and Sex Differences. Sci. Rep..

[16] Hansen M., Kongsgaard M., Holm L., Skovgaard D., Magnusson S.P., Qvortrup K., Larsen J.O., Aagaard P., Dahl M., Serup A. (2009). Effect of Estrogen on Tendon Collagen Synthesis, Tendon Structural Characteristics, and Biomechanical Properties in Postmenopausal Women. J. Appl. Physiol..

[17] Eby S.F., Cloud B.A., Brandenburg J.E., Giambini H., Song P., Chen S., LeBrasseur N.K., An K.-N. (2015). Shear Wave Elastography of Passive Skeletal Muscle Stiffness: Influences of Sex and Age throughout Adulthood. Clin. Biomech..

[18] Chen J., O’Dell M., He W., Du L.-J., Li P.-C., Gao J. (2017). Ultrasound Shear Wave Elastography in the Assessment of Passive Biceps Brachii Muscle Stiffness: Influences of Sex and Elbow Position. Clin. Imaging.

[19] Brooks S.V., Faulkner J.A. (1994). Skeletal Muscle Weakness in Old Age: Underlying Mechanisms. Med. Sci. Sports Exerc..

[20] Miyamoto N., Hirata K., Inoue K., Hashimoto T. (2019). Muscle Stiffness of the Vastus Lateralis in Sprinters and Long-Distance Runners. Med. Sci. Sports Exerc..

[21] Csapo R., Malis V., Sinha U., Du J., Sinha S. (2014). Age-Associated Differences in Triceps Surae Muscle Composition and Strength—An MRI-Based Cross-Sectional Comparison of Contractile, Adipose and Connective Tissue. BMC Musculoskelet. Disord..

[22] Watanabe S., Sato K., Hasegawa N., Kurihara T., Matsutani K., Sanada K., Hamaoka T., Fujita S., Iemitsu M. (2015). Serum C1q as a Novel Biomarker of Sarcopenia in Older Adults. FASEB J..

[23] Barry B.K., Carson R.G. (2004). The Consequences of Resistance Training for Movement Control in Older Adults. J. Gerontol. A Biol. Sci. Med. Sci..

[24] Janssen I., Heymsfield S.B., Wang Z.M., Ross R. (2000). Skeletal Muscle Mass and Distribution in 468 Men and Women Aged 18-88 Yr. J. Appl. Physiol..

[25] Abe T., Thiebaud R.S., Loenneke J.P., Loftin M., Fukunaga T. (2014). Prevalence of Site-Specific Thigh Sarcopenia in Japanese Men and Women. Age.

